# ScabyNet, a user-friendly application for detecting common scab in potato tubers using deep learning and morphological traits

**DOI:** 10.1038/s41598-023-51074-4

**Published:** 2024-01-13

**Authors:** Fernanda Leiva, Florent Abdelghafour, Muath Alsheikh, Nina E. Nagy, Jahn Davik, Aakash Chawade

**Affiliations:** 1 Department of Plant Breeding, Swedish University of Agricultural Sciences (SLU), P.O. Box 190, 23422 Lomma, Sweden; 2 INRAE, Institut Agro, ITAP, University of Montpellier, 34196 Montpellier, France; 3 Graminor Breeding Ltd., Hommelstadveien 60, 2322 Ridabu, Norway; 4 Department of Plant Sciences, Norwegian University of Plant Sciences, 1433 Ås, Norway; 5 Department of Fungal Plant Pathology in Forestry, Agriculture, and Horticulture, Norwegian Institute of Bioeconomy Research (NIBIO), Høgskoleveien 8, 1431 Ås, Norway; 6 Department of Molecular Plant Biology, Norwegian Institute of Bioeconomy Research (NIBIO), Høgskoleveien 8, 1431 Ås, Norway

**Keywords:** High-throughput screening, Imaging

## Abstract

Common scab (CS) is a major bacterial disease causing lesions on potato tubers, degrading their appearance and reducing their market value. To accurately grade scab-infected potato tubers, this study introduces “ScabyNet”, an image processing approach combining color-morphology analysis with deep learning techniques. ScabyNet estimates tuber quality traits and accurately detects and quantifies CS severity levels from color images. It is presented as a standalone application with a graphical user interface comprising two main modules. One module identifies and separates tubers on images and estimates quality-related morphological features. In addition, it enables the extraction of tubers as standard tiles for the deep-learning module. The deep-learning module detects and quantifies the scab infection into five severity classes related to the relative infected area. The analysis was performed on a dataset of 7154 images of individual tiles collected from field and glasshouse experiments. Combining the two modules yields essential parameters for quality and disease inspection. The first module simplifies imaging by replacing the region proposal step of instance segmentation networks. Furthermore, the approach is an operational tool for an affordable phenotyping system that selects scab-resistant genotypes while maintaining their market standards.

## Introduction

Potato is the third most important commodity in the world and represents an essential energy source for human consumption. Potato tubers are processed to provide food, starch, crisps, food additives, and beverages and used for some pharmaceutical products^1^. There is a demand for high-quality tubers that fulfill standards in appearance, size, shape, and flesh or skin color^2^. Regardless of the cultivar, it is essential to guarantee undamaged, appealing, and healthy tubers. Yet these characteristics are challenging to obtain in the context of climate change^3^. Most tuber disorders result from the interaction between environmental conditions, cultivation systems, storage, harvest, or transportation. During growth and handling operations, tubers can sustain various mechanical damage. Likewise, numerous bio-aggressors degrade potato quality and represent a critical threat to their marketability^4,5^.

Such is the case of the common scab (CS) bacterial disease, one of the most important blemish diseases caused by a pathosystem of soil-borne, gram-positive bacteria of the genus *Streptomyces*. The symptoms appear as superficial scab lesions or deep-pitted lesions, downgrading the harvest and resulting in significant economic losses for the growers. Only a few of the several hundred described species are known to be pathogenic to the crop. According to Braun et al.^6^, the two most abundant common scab-causing bacteria in Europe are *Streptomyces turgidiscabies* and *S. europaeiscabiei*. The resistance mechanism to CS is not yet well defined and is still under study. Potato breeders attempt to mitigate the disease spread by developing resistant genotypes that can satisfy both the field and market requirements^7^. Different potato varieties have been recognized to have high levels of resistance to CS under field screenings. Quality assessments of tubers as well as disease severity are generally conducted by visual scorings or manual measurements^8,9^. Although these methods have provided valuable information for selecting desirable genotypes, they are imprecise, time-consuming, and subjective.

On the other hand, digital image processing has improved the consistency and accuracy of plant traits assessment by diminishing the variability caused by human bias^10^. Previous reported studies have evaluated tuber shape, size, and color^11–14^, with accuracies ranging from 70 to 94% compared with caliper measurements and human scorings. Although the results show high accuracies, some challenges remain, such as the lack of user-friendly tools, automation, or adaptation to low-cost and high-throughput phenotyping^15^.

Similarly, some approaches have been reported to assess potato tuber defects^16,17^. Samantha et al.^18^ proposed a method to detect CS based on image analysis in the RGB (red, green, and blue) color space. However, the method uses a series of filters and an unsupervised classifier that is very sensitive to changes in image acquisition conditions^19^. In the range of infrared wavelengths, it has been shown that it is possible to discriminate between infected and asymptomatic areas. Despite the high correlation with the standard severity measurements, the equipment to measure diffuse reflectance is costly and requires seasoned staff to perform acquisitions. Dacal-Nieto et al.^20^ presented a non-destructive approach using hyperspectral imaging combined with supervised classifiers to identify areas affected by CS. The results showed an accuracy of 97.1%, clearly distinguishing the severity levels. However, the method requires a special system to acquire the images that lack operability to be implemented in a breeding context.

In the past decade, deep learning (DL) techniques, especially based on Convolutional Neural Networks (CNNs), have become state-of-the-art approaches in pattern recognition, including plant disease detection and scoring^21,22^. CNNs generate visual representations hierarchy, which is enhanced for a particular task, especially for image recognition and classification that has proved to yield accurate and robust models^22^. They require a training set to calibrate a model with a set of biases and weights corresponding to the target that it was designed for. Among their advantages is that CNNs can process new data and identify significant features with minimal human supervision and tuning. In the case of potato tubers, Oppenheim et al.^4^ proposed a method based on CNN to identify tuber diseases from patches of grayscale images, achieving discrimination accuracies of over 90%. However, the sole identification of diseases is not sufficient to select varieties. An additional scoring of severity levels provides a finer insight into their relative resistance. Thus, developing a robust, user-friendly, and automated imaging method to assess CS infections and tuber morphology is highly valued.

Therefore, this study answers three objectives. The first is to evaluate tuber morphology traits of potato tubers, providing insight into tuber quality for the market. The second is to detect and quantify the level of severity of CS using CNNs. The third is to develop a fully automated and user-friendly application combining the two previous objectives.

## Materials and methods

### Plant material

**Figure 1:**
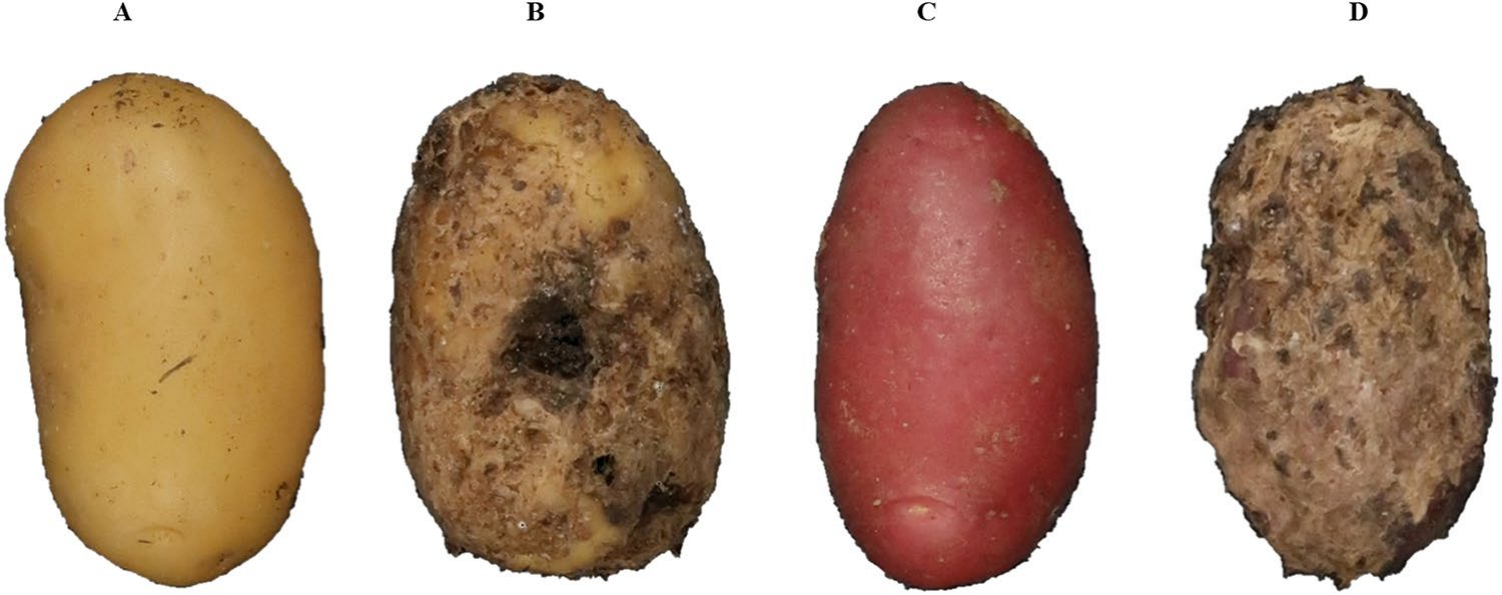
Tile images were used as a training set for the deep learning process. The tiles correspond to a whole potato tuber. (**A**) and (**C**) correspond to a yellow and a red tuber respectively with no symptoms of common scab (CS); (**B**) and (**D**) correspond to a yellow and red tuber respectively with characteristics of severe CS infection, shown as brown spots and lesions on the skin. Scab symptoms can range from a few small lesions on the surface of the tuber to deep and open scab ulcers, covering most of the tuber surface.

Samples of potato tuber were collected from two sources: (1) from Graminor’s core collection grown in field experiments at Ridabu, Norway, from 2019 to 2022, and (2) from a greenhouse inoculation experiment in which 840 interrelated potato lines were planted in sterile peat soil infected with a mixture of three *S. europaescabiei* strains from the NIBIO collection of plant pathogens (isolate nos. 08-12-01-1, 08-74-04-1, 09-185-2-1). In total, 7200 tubers of yellow and red genotypes were used. The core collection tubers represented different levels of infections naturally occurring in the field. Figure 1 shows four samples of tuber containing the full possible range of infections, from completely healthy to maximum severity.

### Image acquisition

**Figure 2:**
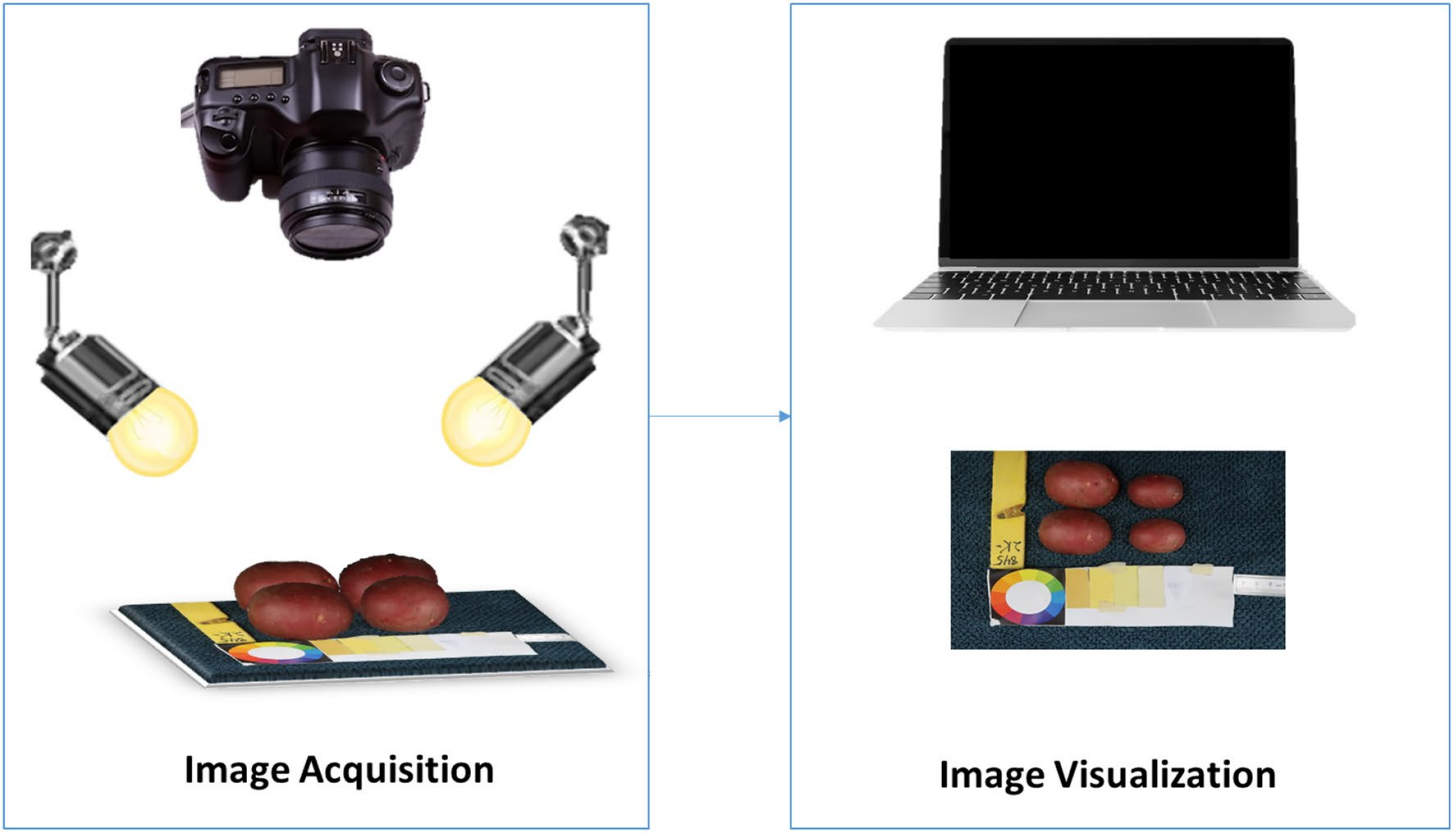
Diagram of the image acquisition protocol.

Tubers were washed, dried, and manually placed in groups of six onto a fiber blue background, along with a 5 cm ruler and a color scale palette for further analysis. Images were captured with a Canon PowerShot G9 X Mark II camera with a lens of 10.2–30.6 mm, 1:2.0–4.9, and a resolution of 20.1 megapixels. The camera was mounted on a Hama photo stand at a top-view angle of 40 cm from the target. The target was uniformly illuminated by daylight bulbs of 85W-5500 K. Camera settings were selected based on the best view of the tubers, ISO 1250, F-stop 1/125, exposure time 1/11, and focal length 10.2 mm. Digital images were stored in JPEG format with a pixel resolution of 7864 × 3648. The size of the tubers varies from 172 to 256 pixels in length. Figure 2 shows an illustration of the image acquisition protocol.

### Database

The database contains 1100 images with 7154 yellow and red tubers. The tubers were categorized into five severity classes, with class 1 being healthy and classes from 2 to 5 that represent increasing severity levels of infection. The classes were attributed based on the area percentage of lesions on the potato tuber skin. A first approximation of the percentage of infected area was obtained in a semi-automated way, using the machine-learning tool Trainable Weka Segmentation (TWS)^23^ as a plugin for the software ImageJ^24^. Manual annotations of 200 images containing 1000 tubers were taken to train a random forest model^25^. The data was segmented into four classes (background, red tuber, yellow tuber, and scab). The segmentation was then taken to a pixel-wise classification where each pixel was classified as belonging to one of the four classes. This first quick approximation with color analysis was then corrected and validated manually.

### Image and data processing

All the image processing was conducted using Python language^26^ with the package OpenCV (Open Source Computer Vision Library)^27^ for image manipulation and analysis of tuber morphology, and TensorFlow^28^ for the deep learning section; the algorithms developed were automated in a GUI (graphical user interface) that could be run over a single image or a large group of images as a batch.

### GUI

**Figure 3:**
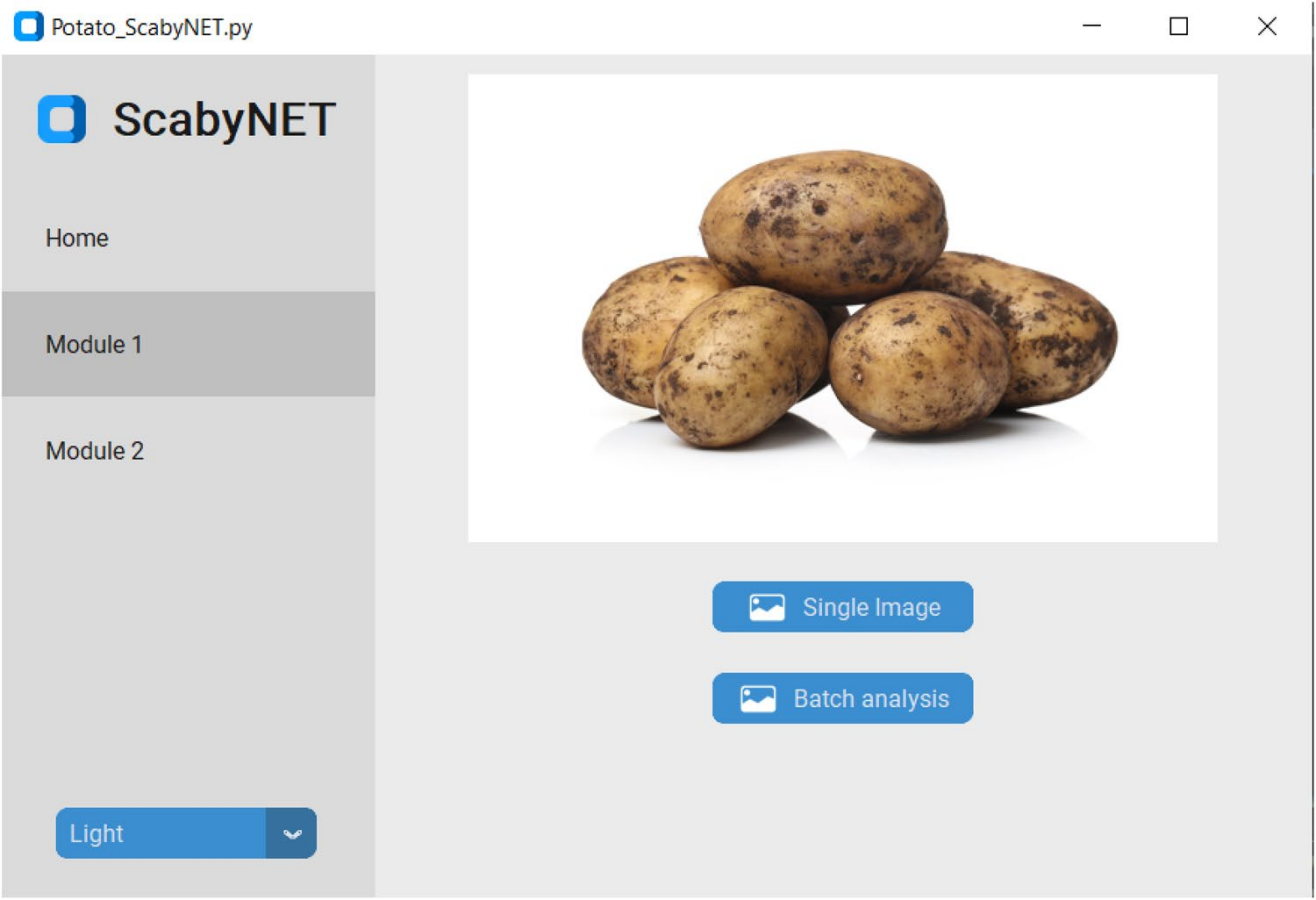
The ScabyNet GUI application with all associated options and functions to evaluate potato tuber images.

The GUI, hereafter called ScabyNet (Fig. 3), was developed in Python using the package Tkinter^29^ and customTkinter^30^. The GUI is user-friendly and contains two main modules and a tab designed as a home window. Modules 1 and 2, corresponding respectively to the estimation of tuber morphology traits and area lesions by CS.

#### Home

The home module contains information about the functionality of ScabyNet, where the user receives instructions on how to use the application.

#### Module 1: morphology features

The morphology module is a fully embedded data processing pipeline that estimates potato tuber morphology characteristics from color images. The module measures for each tuber, length, width, area, length-to-width ratio, circularity, and color values distinguishing between red and yellow tubers. The color analysis is performed in the L*a*b* color space: lightness, a*, and b* chromaticity values for respectively the green–red and yellow-blue axes^31^.

**Figure 4:**
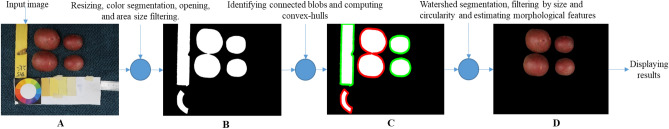
Workflow of the color-morphology module for features extraction, saving, and displaying results. (**A**) Original image to be processed; (**B**) binary image containing the desired and non-desired objects after resized, segmented, and filtered by morphology; (**C**) Identified connected components, non-convex blobs (red) and convex-blobs (green); (**D**) Processed image with segmented tubers.

**Figure 5:**
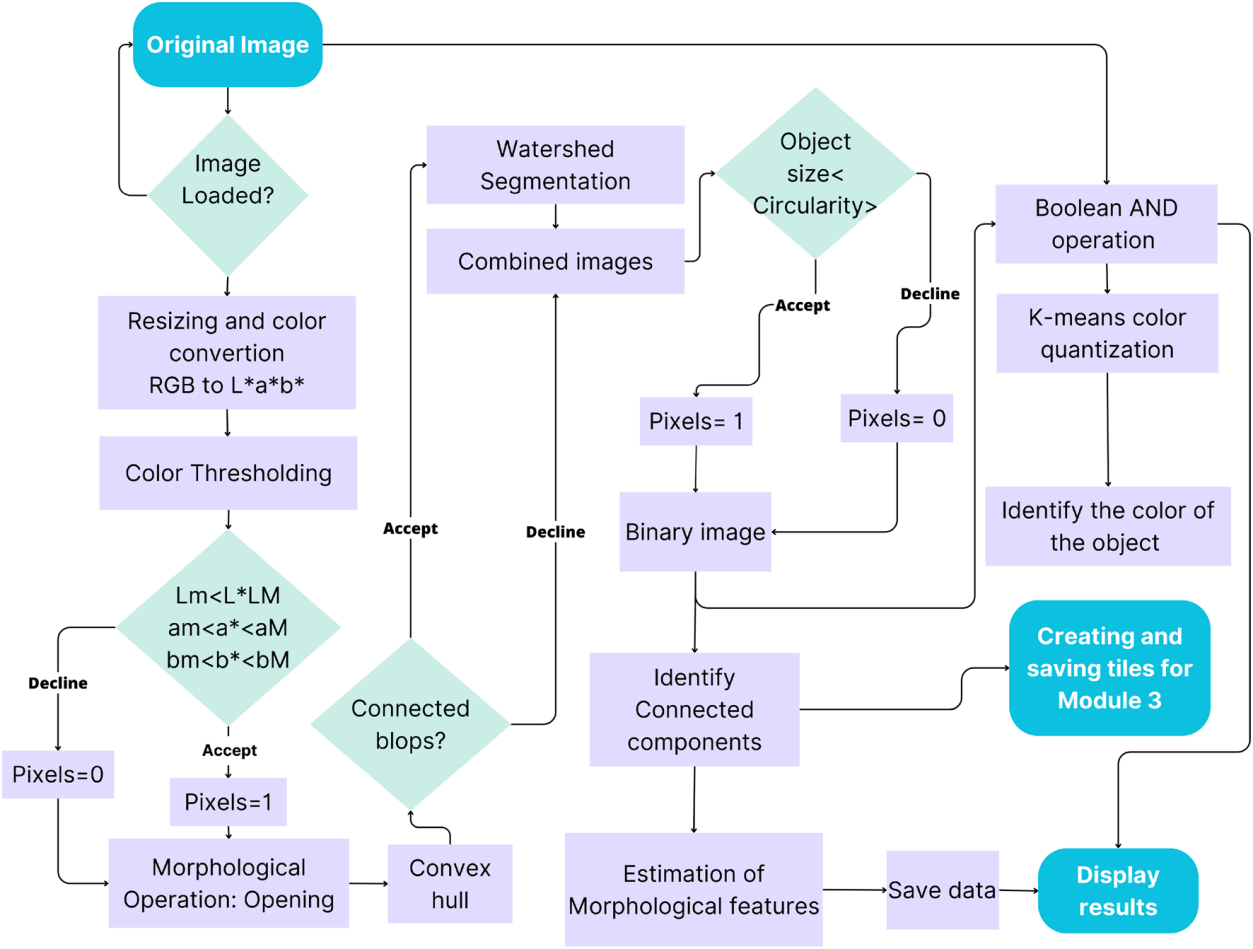
Flowchart of the processing chain, operation algorithms, and outputs for the estimation of tuber morphology features.

The steps in the processing chain of this module are presented in Fig. 4 and described in more detail in the following subsections and the flowchart in Fig. 5.

##### Resizing and color segmentation

To remove the background, facilitate object identification, and decrease computation time, the image size was reduced from 4864 × 3648 to 1459 × 1094 pixels (one-third, conserving proportions). Then, a color conversion was applied from the RGB to the L*a*b* color space, using the features of the OpenCV package. This color representation was chosen because it was designed to approximate human psychovisual representation. A binary filter was applied to remove undesired objects. Each channel of the image was subjected to an examination to determine the adapted threshold. Subsequently, the resulting binary image was used as a mask for the original one.

##### Morphological operation: opening

Due to variations in lighting intensities, drops of shadows, and reflections, some objects in the image contained gaps. They were corrected with the flood-fill algorithm^32^, ensuring object integrity in the image. However, despite this correction of the gaps, some artifacts remained on the image. To discard them, a morphological opening operation was applied^33^. The opening consists of removing pixels on the object boundaries (erosion), then adding pixels to the new boundaries (dilation) on the resulting image. In both cases, the same 5 × 5-pixel square kernel was used as a structuring element. This structuring element identifies the pixel to be processed and defines the neighborhood of connected components based on this binary information. As a result of the opening, the small objects were removed from the image while the shape and size of the tubers were preserved.

##### Identifying connected blob components

Once the segmentation and color reduction was applied, the next step was to identify the tubers. In some cases, tubers were found to be placed too close to each other in the image. Thus, they were detected as a single component. A distinction between connected and disconnected components was performed based on convexity criteria to solve this issue. The operation works in two steps, finding contours, then computing their convex hull^34^. Convex objects i.e. individual tubers were copied and kept apart (noted image A) meanwhile objects corresponding to connected blobs, i.e., joint tubers (noted image B) were submitted separately to a segmentation process to split the connected blobs into the correct individual tubers.

##### Segmenting with watershed transformation

The image containing only the connected blob components (image B), was processed with watershed transformation to split the blobs into individual tubers and obtain the correct tuber count and morphology. The watershed transformation is based on topographic distances. It identifies the center of each element in the image using erosion, and from this point to the edges of the object, it estimates a distance map. Then, this area “topological map” is filled according to the gradient direction, as if it were filled with water. In this way, all connected components are separated (noted image C)^35,36^. Subsequently, image c was combined with image A to gather all the identified individual tubers in only one image (D).

##### Filtering by size and circularity

Once the objects were whole, the tubers were isolated from the non-targeted objects (ruler, color scale palette, genotype serial tag, etc.). For this purpose, a filter was first applied according to the object’s area and then according to the circularity based on Eq. (1) given by Wayne Rasband^24^. After inspecting the area of tubers and their circularity, the min and max values were determined. Only objects of an area between 11,000 px^2^ and 104,000 px^2^ and of circularity higher than 0.7 were retained.

##### Estimating morphology features

Tubers were identified and labeled with an ID, then for each one, the following parameters were measured: area, perimeter, length, width, length-to-width ratio, and circularity. Afterward, to provide a visual representation of the processed input image, the original image was masked with the results from the size and circularity filter, leaving only the tubers. The following formula was used to calculate circularity:

1 $$Circularity = \frac{{\left( {4{ } \times \pi \times { }area} \right){ }}}{{Perimeter^{2} }}$$

##### Identifying tuber skin color

The tubers contained a complex color spectrum corresponding to variations of the skin, buds, lenticels, mechanical damage, common scab symptoms, and other possible defects. To overcome this problem, color identification was performed using a K-means color quantization^37^, aiming both at facilitating the identification and reducing computation time. The process consists of reducing the number of colors in an image from 256 × 256 × 256 possible values in the 8-bit RGB color model to the desired number of colors but preserving the important information of it. In this case, three colors were selected, (background and the two considered colors for tubers). Based on these values (clusters), the centroids were determined. Then the color was determined, according to the minimum Euclidean distance between all the respective colors present in the image to the three cluster centroids. Several repetitions were performed until the centroid of clusters did not show changes and the distance between the centroids and the color objects was minimal while the distance between centroids was maximal. Subsequently, the image was segmented into three colors and an 8-bit value was given to the respective object, ‘0’ to the background, ‘1’ to the red tuber, and ‘2’ to the yellow tuber.

##### Displaying results

When analyzing an individual image the results are directly displayed on the screen. A window with the image containing only the previously labeled potato tubers, and another window with a table containing the estimations of morphology and color features. On the other hand, when selecting a batch of images, the results are saved in a folder named ‘Results’ in the same source directory given by the user. The folder contains the processed images with the potato tubers labeled and a CSV file with all the measurements linked to the respective IDs.

#### Module 2: common scab detection

##### Deep learning

The deep-learning module processes individual tiles of fixed size (172 × 172 pixels) representing an individual tuber. The tiles contain the segmented tubers without background, resulting from the morphology module's output.

##### Convolutional neural network architecture

A benchmark including six common architectures of CNN was conducted to model and predict the severity level of scab infection: VGG16, VGG19, ResNet50V2, ResNet101V2, InceptionV3, and Xception. These architectures were developed for different object recognition applications, including plant and disease classification, and ranked among the best performing in the deep-learning challenges^38^. A table comparing their characteristics is described in Table 1.

**Table 1:** Comparison of the six CNN architectures used in the benchmark.

CNN Architectures	Description	References
VGG16 and VGG19	Contain respectively 16 and 19 weighted layers. Simple and computationally efficient and easy to adapt to relatively small datasets	^40^
Residual Network (ResNet) 50V2 and 101V2	Introduce residual connections, to process smaller objects while keeping the ability to learn complex features. 50 and 101, respectively denote the number of layers in the networks	^41^
InceptionV3	Based on sub-structures to learn features at multiple scales simultaneously. Adapted to capture some differences between patterns that share many common features	^42^
Xception	Utilizes depth-wise separable convolutions, a type of convolutional operation that separates the spatial filtering and channel-wise processing of the input. Reduce the number of parameters to optimize the training complexity while retaining the important features	^43^

**Figure 6:**
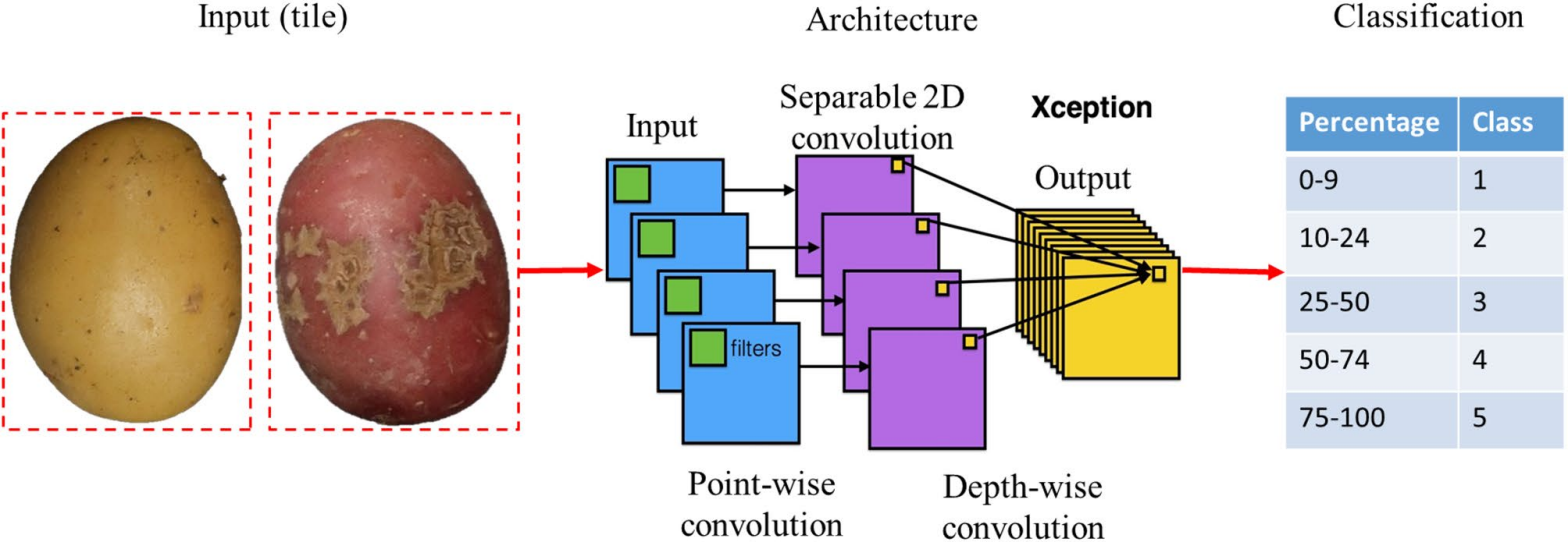
Schematic of module 2 for the detection and quantification of CS severity using deep learning with the ResNet architecture.

Different training strategies were compared and the training parameters were optimized according to the following criteria: minimizing the false positive rate of the infected classes in the health class and maximizing the separability between the minor and severe infection classes. The compared strategies were transfer learning and fine-tuning (Table 2). For both strategies, the networks are initialized with the weights resulting from the training on the ImageNet dataset containing 1.2 million images in 1000 classes such as “cat”, “dog”, “person”, and “tree”, among others^39^. In addition, we evaluated the robustness of the model with standard metrics (loss and accuracy). A schematic overview of ScabyNet-module 2 is shown in Fig. 6.

**Table 2:** Comparison of the two machine learning models for CNN used in the benchmark.

Strategy to transfer learning	Description	Advantages	References
Transfer learning (generic)	Use a pre-trained model that previously learned generic features on wide and diverse databases and adapts the classification to a new problem	Save training time, does not require a lot of new annotated data	^44^
Fine-tuning	Use the weights of a pre-trained network for a new task, and new classes from new data. The first layers of the network, which extract generic features common to many objects are fixed, while the last layers extract more complex and specific features, which are updated according to the new data	Allow the network to leverage optimal parameters learned from a robust database and fit it with a lesser cost for the classes of the new application	^45^

Generally, the complete training of a CNN is computationally intensive and requires a substantial amount of annotated data. These data are usually gathered from multiple collaborative projects. In the case of new applications, where less data is available, it is common to use a pre-trained network from public databases and adapt them to the specific application.

#### Visual inspections and manual measurements

##### Manual measurements for morphology traits

Tubers were measured manually using the ImageJ software^24^, using the 5 cm ruler placed at the bottom of the images as a scaling reference. Each potato was selected, and using the option “line” from the toolbox, the length and width were measured, then using these two parameters the length-to-width ratio was calculated.

##### Expert scores for disease severity of CS

The severity levels of CS are usually assessed visually and scored by an expert evaluating two parameters. First, the surface area covered with scab lesions, and second, the severity level, i.e., how deep the scab lesion is observed. The surface area covered is rated on a scale from 0 to 9, where 0 corresponds to no scab lesions on the surface, and 9 corresponds to about 100% of the surface area covered with lesions. The depth of the scab lesion is rated on a scale from 1 to 3, where 1 = superficial lesions, 2 = raised lesions, and 3 = deep lesions, the most severe coverage. Here, only the surface area was used and the expert scoring in ten grades (or severity classes) was transformed into a five classes severity scale.

##### Classes for CS

**Figure 7:**
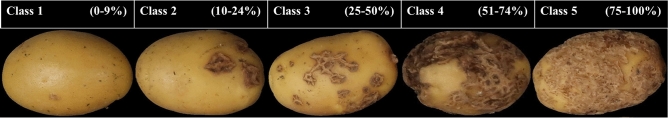
Images of the different severity levels of CS on yellow tubers. The rating of disease severity ranged from 0 to 100%. Scoring was based on the surface coverage of CS on the tuber.

Potato tuber images were visually selected and classified in five classes, depending on the level of severity of CS on the surface area. Class 1 corresponds to 0–9%, class 2 to 10–24%, class 3 to 25–50, class 4 51–74%, and class 5 to 75%–100%. Figure 7, shows the scoring scale with corresponding images.

### Statistical analyses

Statistical analyses were performed using R version 4.1^46^ and Python version 3.9^47^. To evaluate module 1, the Pearson correlation coefficient was computed between the ground truth (manual measurements of the tubers), and the results obtained respectively with ImageJ and ScabyNet. For module 2, the two training strategies fine-tuning and transfer learning were compared. To ensure the reliability of the benchmark, the dataset (7154 potato tubers) was split into training, validation, and testing sets. By employing the function “Random Split” from Scikit Learn^48^, the main dataset was fractionated into 70% for the training set and the remaining 30% as a testing set. Subsequently, the training set was divided again using the same function to perform cross-validation, into 70% for the training set and the remaining 30% as a validation set. The results were compared with expert scoring in order to verify the accuracy of module 2.

### Research involving plants

All the methods employed regarding plant materials followed the strict rules of the Swedish Agricultural University which are in accordance with all international standards, including those in the policies of Nature.

## Results

ScabyNet is a user-friendly application that contains two main modules and a home tab dedicated to providing information on how to use the application. Modules 1 and 2 were designed to process images for morphology traits and CS severity. In both cases, an individual image containing any number of potato tubers or a batch of images could be analyzed. For the first case (individual image), the user selects the image file and after the analysis, the resulting image is displayed on the screen with the morphological features in a separate table. Then, the user decides whether to save the results or not. In the second case (a batch of images), the user selects the source folder containing the images to be analyzed, and in the same folder, a subfolder named “Results” is automatically created in the root of the data, and corresponds to the storage of the resulting processed images and the CSV file with the data information.

### Module 1: morphologic features

#### Performance test

To assess the consistency of the morphologic features analysis, a dataset of 100 randomly selected images containing different numbers, shapes, and sizes of potato tubers was analyzed. In total 4735 tubers were processed.

##### Tuber size

**Figure 8:**
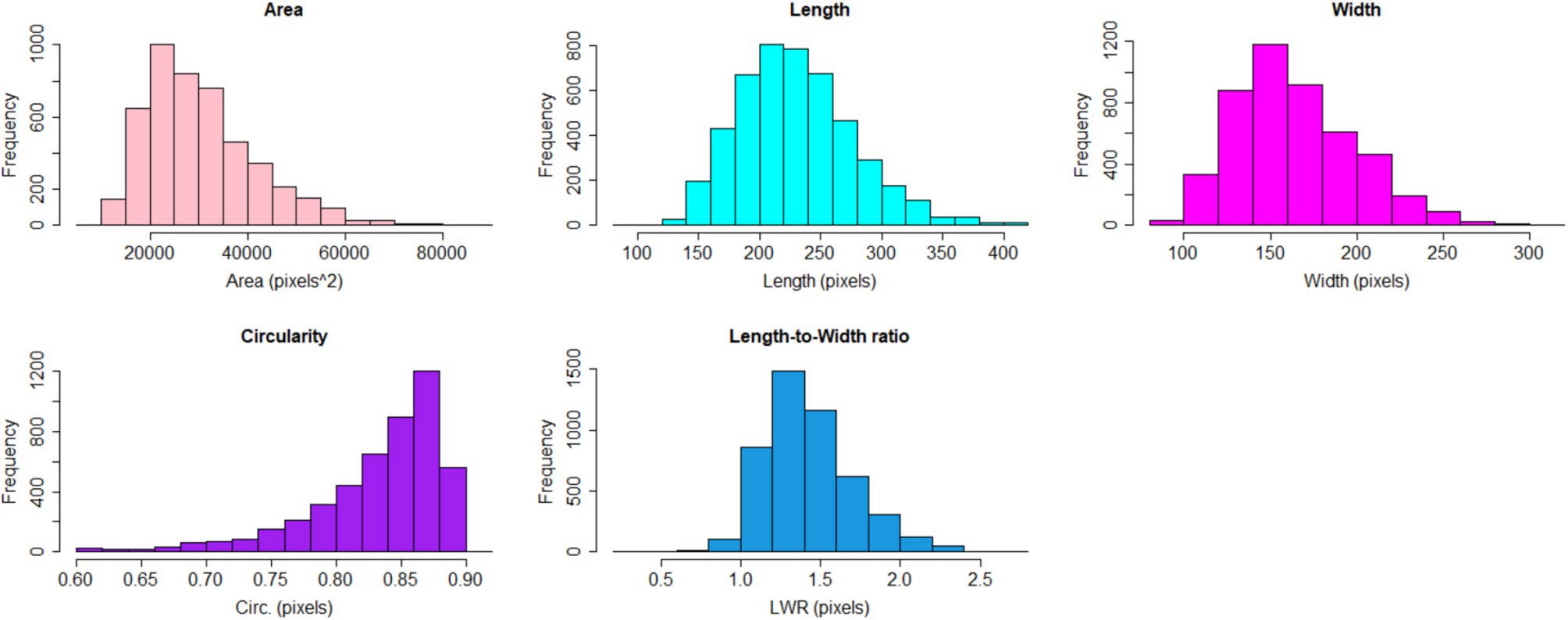
Frequency distribution of the different morphological traits measured with ScabyNet module 1, including area, length, width with the respective units, circularity (Circ.), and length-to-width ratio (LWR).

The results obtained by ScabyNet were compared with ground truth data and a method proposed for ImageJ^24^. A medium–high correlation was observed with the ground truth and ScabyNet (> 0.84; Table 3). For the case of correlation between ScabyNet and ImageJ, the results show a high correlation (> 0.88; Table 3). Hence, ScabyNet provides a robust and reliable approach to evaluate tuber size features like the ones here described. Figure 8 shows the frequency distribution of all the morphological traits measured with this module. All the traits showed an almost symmetrical Gaussian distribution, except for the circularity that showed a skewed left.

**Table 3:** Pearson correlation coefficients of the three methods for estimating different tuber size parameters, ScabyNet, ImageJ, and Ground truth measurements.

Morphology parameter	ScabyNet vs ImageJ	ScabyNet versus ground-truth
Area	0.86	NA
Width	0.92	0.85
Length	0.86	0.79
L/W	0.86	0.89
Circularity	0.90	NA

##### Time efficiency

Images were processed in a computer with an Intel(R) Core(TM) i7-8650U CPU processor at 1.90 GHz 2.11 GHz. Time was recorded for all the steps required to analyze an image, starting with image acquisition and ending with saving the data. A complete analysis is described in the following subsections.

- *Image acquisition:* Establishing the image acquisition protocol
  Organizing the shooting place with the illumination panels, setting up image parameters, and placing the camera in the stand at 40 cm took 10 min. This step is done only once during the analysis. Then, placing the previously washed tubers on the background took 2 min in batches of six tubers. The time for capturing the image took less than 5 s. The time taken for cleaning the potatoes was not accounted for because it was already required before performing visual inspections of the tubers.
- *Image processing*: Executing ScabyNet GUI

Estimating the time approximately that a user took to analyze an individual image with 6 potato tubers was around 4 s. The time of selecting the image file depends on the accessibility of the file. A more detailed inspection was performed with images containing different numbers of potato tubers (Table 4A). The results showed that the analysis of one image containing up to 12 potato tubers lasts between 1 to 3.5 s. For a batch, the time varies depending on the number of images to analyze. A time recording was performed with a dataset of 100 images with 4735 potato tubers (Table 4B).

**Table 4:** Performance test using ScabyNet, A) for an individual image, showing the time taken to analyze a different number of tubers, from 0 to 12 tubers. B) For a batch of images, showing the time taken to analyze a different number of images containing 6 tubers each one of them.

(A)	No. of Tubers	Time (s)	(B)	No. of images	Time (s)
	2	2.47		10	20.45
	4	2.53		20	21.51
	6	2.90		40	25.10
	8	3.01		60	26.80
	10	3.19		80	28.19
	12	3.38		100	31.38

### Module 2: scab detection using deep-learning

The dataset composed of 7154 individual tuber tiles, from both red and yellow potato varieties was randomly divided into two main subsets. A learning set, composed of 70% of the data and used to calibrate and optimize models, and a test set, containing the remaining 30%, is used to assess the performance of the models on independent data.

During the training phase, a cross-validation is performed for which the learning set was itself divided into a training set, which constituted 70% of the learning set data, and a validation set with the remaining 30% of data.

### Training steps

**Figure 9:**
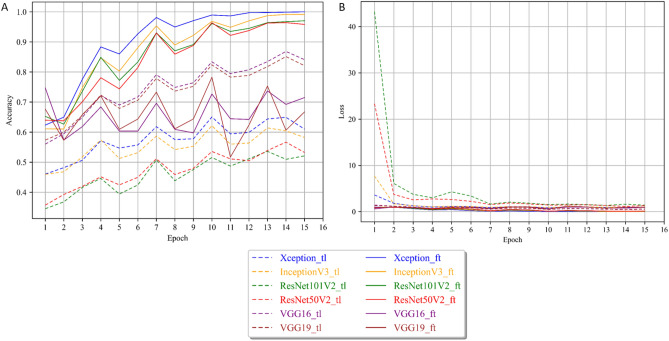
Training performance for the transfer learning and fine-tuning models. (**A**) Accuracy curve and (**B**) Loss function curve.

**Figure 10:**
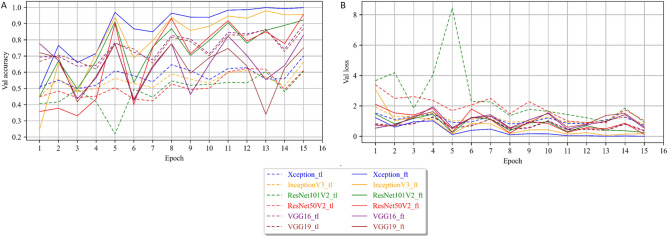
Validation performance for the transfer learning and fine-tuning models. (**A**) Accuracy curve and (**B**) Loss function curve.

Figure 9 represents respectively the training accuracy (A) and the training loss (B). Figure 10 represents the validation accuracy (A) and the validation loss (B).

The models trained with the transfer learning strategy are denoted with “_tl” and displayed as dashed curves, while the ones trained with fine-tuning are denoted with “_ft” and displayed as continuous curves. The different models were trained according to the parameters presented in Table 5, respectively to the architecture types and the training strategy.

**Table 5:** Parameters used for the training of the different models.

Training strategy	Architectures	VGGs	ResNets /Inception/Xception
Transfer learning	Epochs	15	15
	Batch size	64	64
	Learning rate	10^–3^	10^–3^
Transfer learning with Fine-tuning	Epochs	15	15
	Batch size	64	64
	Number unfrozen layers	5	10
	Learning rate frozen	10^–3^	10^–3^
	Learning rate unfrozen	10^–4^	5 × 10^–5^

All architectures showed a typical learning behavior, with increasing accuracy combined with a progressive decreasing loss at each epoch. Generally, the fine-tuning strategy presents significantly better performances than the transfer learning strategy for the deeper and more sophisticated architectures (ResNet50V2 and ResNet101V2, InceptionV3, and Xception), both in the training and validation. On the other hand, the simpler VGG networks (VGG16 and VGG19) showed better performances in fine-tuning (Fig. 9). With the transfer learning strategy, the ResNet architectures cannot be trained for the CS application. Their respective training accuracy barely exceeded 50% after 15 epochs, and the training loss did not decrease from 7 epochs. This means the produced model was equivalent to random decisions and did not include any new information. The validation performances showed the same behavior and confirmed that the training of these architectures in transfer learning failed with the available data. Similarly, InceptionV3 and Xception also showed poor transfer learning performance, with maximum accuracies of just over 60%. The two models quickly stagnated after a few iterations, and their weights did not exhibit any significant change after 6 epochs. The validation’s respective accuracy and loss showed the same poor performances. For VGG16 and VGG19, the training accuracy reached over 80% accuracy after 10 epochs and reached its maximum at 14 epochs with 85% and 86% accuracy, respectively. However, when looking at the validation results, performances became increasingly unstable, with drops of up to 10% accuracy between successive epochs. We can then attribute their relatively good performances in training only to a form of overfitting. Similarly, in fine-tuning, the VGG architectures seemed very unstable, as shown by their training and validation accuracies, which shifted substantially between epochs. In addition, they never reached above 80% accuracy.

Eventually, only four models, InceptionV3, Xception, ResNet50V2, and ResNet101V2, reached performances over 90% accuracy, all in fine-tuning. However, these results proved to be only consistent for InceptionV3 and Xception as it is shown by the difference between training and validation behavior for the ResNets (Fig. 10A). Likewise, only InceptionV3 and Xception showed stable results as shown by the validation accuracy and loss (Fig. 10A,B). In addition, these two models showed no sign of overfitting, as shown by the consistent increase in accuracy coupled with decreasing loss after reaching more than 95% accuracy (a more detailed view of the losses can be found in Supplementary Fig. 1).

Ultimately, the most accurate and stable model was Xception trained in fine-tuning. The results showed that this architecture with this training strategy reached a stable accuracy of over 95% after 10 epochs and consistently improved until reaching 99% accuracy on the validation set while keeping a low loss.

#### Test step

Tables 6, 7, and 8 present the confusion matrices of the test data for the Xception models trained in fine-tuning after 15, 12, and 10 epochs, respectively, and the corresponding precisions detailed for each class. The actual classes are presented in the rows versus the predicted classes in the columns. In total, 2146 tubers were tested with the following repartition into the classes: 317 class 1 (healthy), 712 class 2, 591 class 3, 351 class 4, and 174 class 5. The test set was sampled randomly, respecting the proportion of classes presented in the complete dataset. At 15 epochs (Table 6), only 6 tubers out of 2146 were misclassified, resulting in accuracies above 99% for all classes. At 12 epochs (Table 7), the precision was above 95% for all classes except class 5, i.e. the tuber most severely affected by CS, for which the precision was 92%. At 10 epochs (Table 8), the precision was above 90% for all the classes except class 4. In this case, there was confusion between class 3 and class 5 with some tubers belonging to both classes. This mostly happened in class 4 for 10 epochs. The test of Xception, trained in fine-tuning with the last 15 layers unfrozen, shown on independent data, increased performances consistent with the training. The model discriminated perfectly healthy and lightly CS-infected tubers from the severe forms. Even the moderate symptoms (classes 3 and 4) can be distinguished with the optimal model. This means that optimally trained with adequate parameters and strategy, Xception can easily distinguish infection classes describing a 10% to 25% difference in infected areas.

**Table 6:** Confusion matrix of the test data predicted for the Xception architecture using the model fine-tuning with 15 epochs.

Xception_ft_15	Predicted class						
Actual class		Healthy 1	CS 2	CS3	CS4	CS5	Total
	Healthy 1	317	0	0	0	0	317
	CS 2	1	710	1	0	0	712
	CS 3	0	2	590	1	0	593
	CS 4	0	0	0	350	1	351
	CS 5	0	0	0	0	173	173
	Total	318	712	591	351	174	2146
	Precision	> 99%	> 99%	> 99%	> 99%	> 99%

**Table 7:** Confusion matrix of the test data predicted for the Xception architecture using the model fine-tuning with 12 epochs.

Xception_ft_12	Predicted class						
Actual class		Healthy 1	CS 2	CS3	CS4	CS5	Total
	Healthy 1	311	8	0	0	0	319
	CS 2	5	698	13	0	0	716
	CS 3	2	5	572	6	4	589
	CS 4	0	1	4	334	9	348
	CS 5	0	0	2	11	161	174
	Total	318	712	591	351	174	2146
	Precision	98%	98%	97%	95%	92%

**Table 8:** Confusion matrix of the test data predicted for the Xception architecture using the model fine-tuning with 10 epochs.

Xception_ft_10	Predicted class						
Actual class		Healthy 1	CS 2	CS3	CS4	CS5	Total
	Healthy 1	309	18	3	0	0	330
	CS 2	5	676	29	4	0	714
	CS 3	3	15	542	35	2	597
	CS 4	1	3	14	285	7	310
	CS 5	0	0	3	27	165	195
	Total	318	712	591	351	174	2146
	Precision	97%	95%	92%	73%	95%	

## Discussion

In plant breeding, tuber quality, in terms of shape, size, and severity level of CS, still relies on manual measurements and low-throughput visual assessments. These approaches are known to suffer from a lack of accuracy and reproducibility on top of being time-consuming and labor-intensive. ScabyNet proposes an image-based method divided into two modules to measure the morphological features of tubers and to assess the severity level of CS. The results of both modules indicated a high correlation between the manual measurements and the visual scoring of the evaluated tubers. Furthermore, a comparison of the applicability of the two modules was properly addressed by evaluating the time and accuracy.

### Module 1

Several studies have been reported to evaluate tuber morphology and had reached high correlations with manual measurements. However, some inconsistencies in outputs can be found in the simpler approaches while the most advanced ones are costly, often impractical, or unsuitable for full-scale trials^12,13,17,33^. Here a low-cost image acquisition and processing approach was implemented, requiring only a simple RGB camera, a static frame, a light panel, and ScabyNet. While comparing with similar approaches^11,12^ greater differences were found. First, the potential of these approaches is limited for images containing a greater variability in tuber shapes and color or containing several tubers or other objects. Second, to be properly processed, images must be acquired with a strict protocol, including using a lightbox^11^. In the same way, as described in^11^, ScabyNet also estimates circularity and LWR, which allow the screening of new varieties for different markets in terms of quality^13,49^. Unfortunately, ScabyNet can only evaluate two-dimensional tuber shapes, which is a limitation in terms describing the real consumer value of tubers. Different studies have been conducted evaluating all the possible views of an object, and a close approach has been reported even to predict diseases based on seed morphological parameters^50^. Using a cost–benefit instrument, Cgrain^51^, it is possible to obtain a full 3D view of the seed and analyzed parameters almost instantaneously. This could provide more detailed shape information compared to 2D imaging in the case of tubers. However, the size of the tubers would need a specific instrument or a 3D imaging platform, which in terms of time and labor will not represent an efficient approach.

Considering tuber shape, it has been found that it can be a complex parameter to evaluate, especially for those with abnormal shapes or really flattened, which depends mainly on the different purposes of the final use. In order to have a standard measurement, the length and the width were set up as the potato was always placed horizontally, but this parameter can change if the potatoes are placed in a vertical position. For this case, there is a step that analyzes if there is any inconsistency between width and length, identifying the longest axis the length, and the shortest one the width, while LWR and circularity are calculated directly with these two parameters.

An important aspect to highlight is that, compared with other approaches, ScabyNet, showed to be robust and accurate, but above all much easier to implement. In terms of comparison with TubAR^12^, despite being a free application for R software^46^, it requires some pre-install packages and program execution requires running several command lines. This means that only seasoned operators are able to use the application. For the case of the approach presented for the software ImageJ^11^, there is only a set of commands to follow but no complete application is proposed, which in the same way only can be applied by seasoned operators.

### Module 2

In module 2, the best-performing model embedded in ScabyNet was the Xception architecture, which reached high accuracy and proved to be robust and stable for the tested dataset and the considered severity classes. The fine-tuning strategy was adapted to disease scoring, as it was a substantially different task from what the CNNs are usually trained for. The sole adaption of the weights in the deep neural network (DNN) part of the network, i.e., the classifier part, was not enough to distinguish between basic severity classes. This means that new weights in the filters were necessary to capture the specific patterns associated with CS coverage on the tubers. The specificities of the Xception architecture enabled to adapt efficiently the model to CS scoring with a reduced dataset. Other tested architectures (except InceptionV3) and training strategies could not provide the same performances or stability. They would either require more data to converge toward the right filters extracting the right features in the cases of the advanced architecture; in the case of the VGGs, they are simply not complex or deep transfer the common features learned from generic data to be applicable to the CS detection and scoring problem. Most likely, the advanced networks would also require deeper training of the CNN part, i.e., consisting in unfreezing more layers and reaching earlier layers in the backpropagation. Consequently, they would be more likely to show instabilities or even overfitting considering the relative size of the available database in agriculture and the ones used to calibrate the pre-trained models.

With the optimal model, some confusions are still possible, mostly between severity levels that are close to each other. A solution to improve the model should be to determine classes as severity profiles rather than based on ranges of infected areas and thus match more the assessment rules of breeders. As no other studies to our knowledge tackle the issue of scoring potato CS with an automated image-based approach, it is not possible to compare the obtained results. A solution to improve both the performances and the sensitivity of the model, i.e. being able to distinguish between finer differences in infection profile, would be to fine-tune more deeply or even retrain the network with a generic plant disease database like “PlantVillage” or “PlantImageAnalysis”^52,53^. These databases contain hundreds of thousands of examples of healthy and infected plants from more than a hundred species, different pathogens, and infected organs. The generic features learned from that should be a better starting point to adapt the network to CS, or even to generalize ScabyNet to various plants and diseases.

## Conclusion

This study proposes a novel application named ScabyNet that combines traditional image processing techniques and deep learning algorithms to estimate potato tuber morphology features and the detection and severity scoring of CS disease. This approach demonstrated operational qualities such as versatility and efficiency in analyzing images of potato tubers of various sizes, shapes, and colors, and with different levels of CS disease severity. The accuracy of ScabyNet was validated through correlation with manual measurements and with a previously established method for measuring potato tuber length and width, as well as visual correlation with disease severity scores. Among six different architectures and two training strategies tested, the one selected for ScabyNet outperformed the others with an accuracy of 99%.

Notably, ScabyNet was developed as a lightweight application that relies solely on CPU computation, enabling greater portability and ease of deployment on a wider range of computing systems. These findings demonstrate that ScabyNet represents a significant advancement in agricultural research, providing an efficient, accurate, and objective method for analyzing tuber morphology features and estimating CS disease severity in potato crops.

In future research, it is planned to extend the applicability of ScabyNet to include additional color ranges of tubers and other potato varieties and incorporate semantic segmentation to achieve higher precision and accuracy in tuber identification. The purpose would be to reach finer levels of discrimination between infection stages and to recognize specific patterns of the symptoms to match better with phytopathology. Furthermore, incorporating additional spectrometric data, such as hyperspectral imaging, may provide further insights into the finer phenomena related to the disease and allow for the detection of early symptoms before they become visible^20^.

### Supplementary Information

Supplementary Figure 1.

## Data Availability

The datasets generated and analyzed during the current study are not publicly available due to being obtained from a commercial breeding program but are available from the corresponding author on reasonable request.
