# Supplementary material for: ScabyNet, a user-friendly application for detecting common scab in potato tubers using deep learning and morphological traits
Source: Sci Rep. 2024 Jan 13;14:1277. doi: 10.1038/s41598-023-51074-4 (PMC10787732; doi:10.1038/s41598-023-51074-4)
Supplement: Supplementary file 1 — Supplementary Figure 1. [file 41598_2023_51074_MOESM1_ESM.docx]

**Supplementary Material**


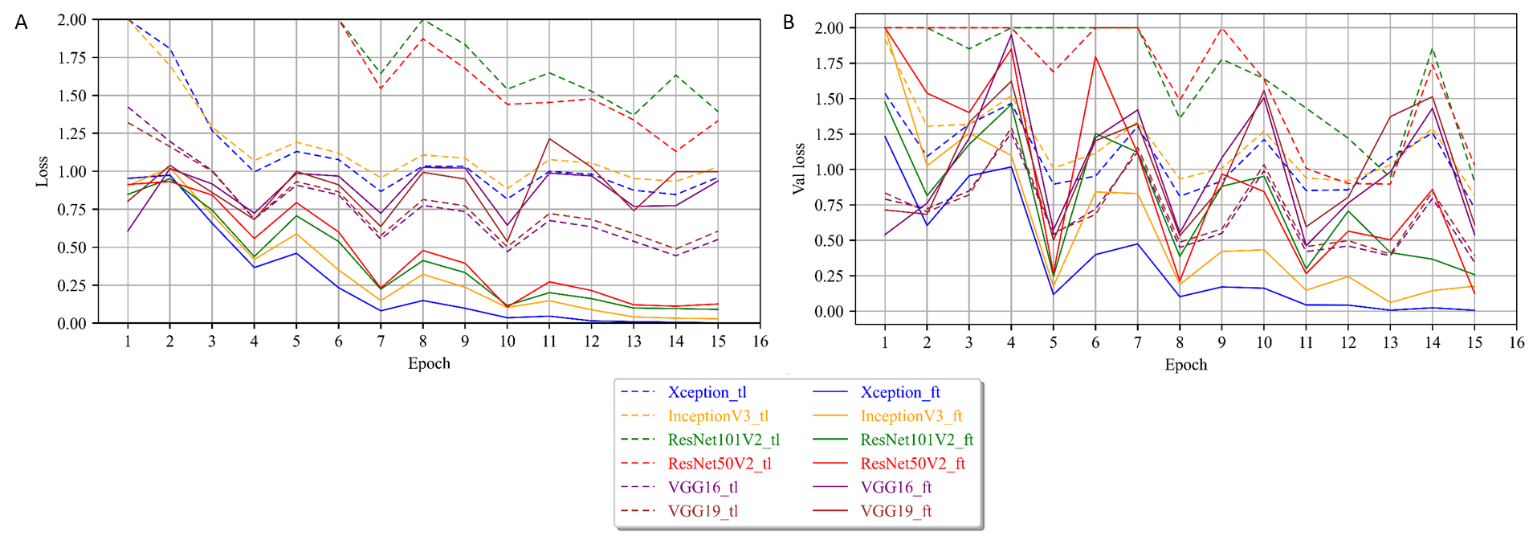


**Supplementary figure 1.** Zoom of the training output, A) training loss and B) validation loss
